# Supplementary material for: Evaluation of Mitochondrial Dysfunction and Idebenone Responsiveness in Fibroblasts from Leber’s Hereditary Optic Neuropathy (LHON) Subjects
Source: Int J Mol Sci. 2023 Aug 8;24(16):12580. doi: 10.3390/ijms241612580 (PMC10454080; doi:10.3390/ijms241612580)
Supplement: Supplementary file 1 [file ijms-24-12580-s001.zip › ijms-2454828-supplementary.pdf]

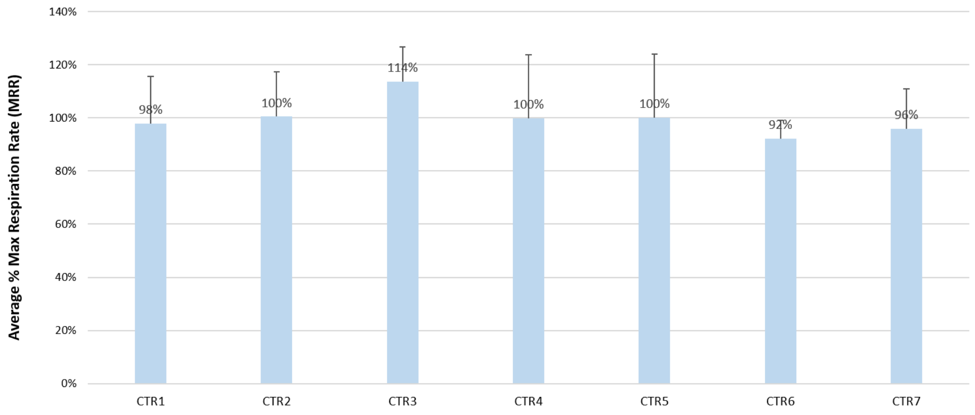

Figure S1. Maximal respiratory rate evaluations in control cell lines. Percentage of maximal respiratory rate (MRR) in 5 fibroblasts cell lines comparted to the two controls CTR1 and CTR2. CTR3-CTR7 fibroblasts were obtained from healthy subjects or unaffected carriers of single heterozygous variant responsible for recessive disorders. Experiments were performed in duplicate, in standard culture media. The average MRR of the 2 CTRs was set as 100%. Each cell line from CTR3 to CTR7 showed non-significant (p-value >0.05) difference compared to the two main controls (CTR1 and CTR2).

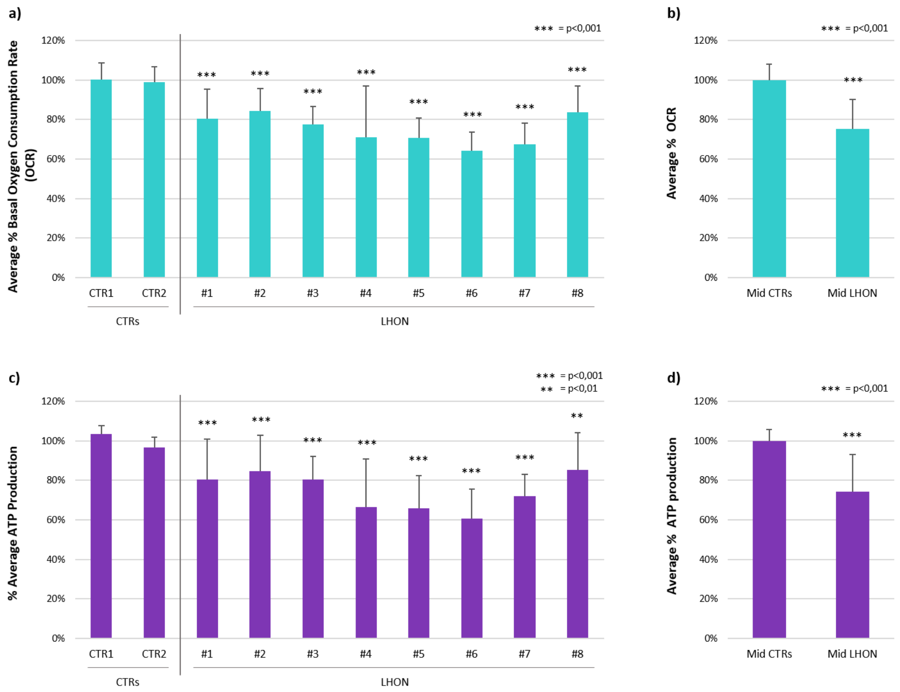

Figure S2. Oxygen consumption rate and ATP production. (a) Percentage of basal oxygen consumption rate (OCR) of 8 LHON fibroblasts cell lines comparted to two controls (CTRs) in standard culture media. The average OCR of the 2 CTRs was set as 100%. (b) OCR mean value in control and LHON cells. (c) Percentage of ATP production rate of 8 LHON fibroblasts cell lines compared to 2 CTRs in standard culture media. The average ATP production of the 2 CTRs was set as 100%. (d) ATP production rate mean value in control and LHON cell lines. (*** = p-value < 0,001; ** = p-value < 0,01).

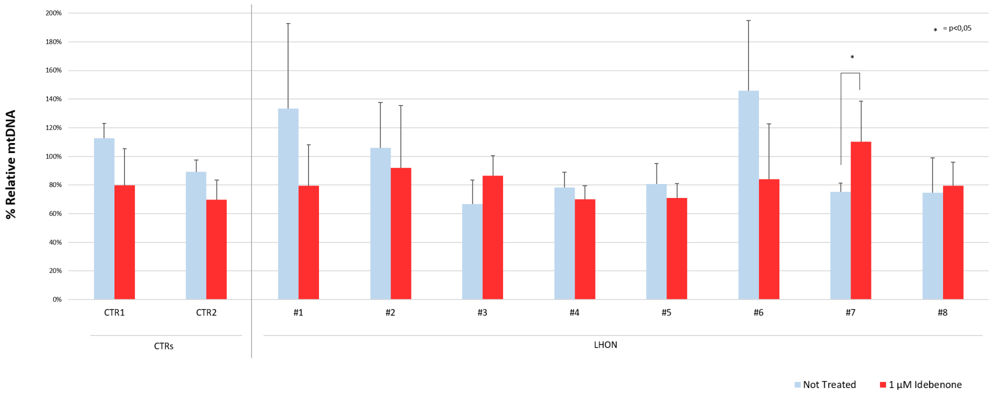

Figure S3. Evaluation of mitochondrial DNA amount in LHON fibroblasts. Percentage of relative mtDNA/nDNA amount before (blue columns) and after (red columns) 48 hours of 1 µM idebenone treatment. The mean value of the ratio mtDNA/nDNA in untreated control cells was set as 100% (* = p-value < 0,05).
